# Quantifying hydrothermal ammonium mobilization from sediment and implications for the marine biosphere: a case study from the Guaymas Basin, Gulf of California

**DOI:** 10.3389/fmicb.2025.1523696

**Published:** 2025-07-16

**Authors:** Nathan Rochelle-Bates, Annabel Long, Graeme A. MacGilchrist, Andreas Teske, Eva E. Stüeken

**Affiliations:** ^1^School of Earth & Environmental Sciences, University of St Andrews, St Andrews, United Kingdom; ^2^Department of Earth, Marine and Environmental Sciences, University of North Carolina at Chapel Hill, Chapel Hill, NC, United States

**Keywords:** hydrothermal, Guaymas Basin, ammonium, *Beggiatoa*, organo-metal complex

## Abstract

Fluid-rock interactions in hydrothermal systems are capable of liberating ammonium (NH_4_^+^) from sedimentary organic material and making it bioavailable for benthic and pelagic microbial communities. Hydrothermal systems in organic-rich sedimentary basins are therefore thought to have played a key role in supplying bioavailable nitrogen to the early biosphere. To place new quantitative bounds on this process, we examined sediments from active hydrothermal systems in the Guaymas Basin, a young oceanic spreading center in the Gulf of California. We analysed four shallow sediment cores that were taken in the Guaymas Basin’s hydrothermally-active Southern Trough. We used a combination of isotopic tracers (δ^15^N, δ^13^C) and elemental abundances to explore nitrogen and metal mobility in buried sediments. We found that ca. 54% of the organically-bound nitrogen is remobilized by active seepage in the top 10 cm of the sediment package within as little as 27–83 yr. Extrapolating these findings over the hydrothermally-active area of the basin yields an ammonium seepage flux of ca. 1.3–4.1 mol/s. In addition, high temperature venting liberates ca. 156–187 mol/s, as estimated from previous data. Assuming biological uptake of hydrothermally recycled ammonium in the water column, these fluxes could support up to 1.3% and 58% of export productivity, respectively. Our data also reveal that the accumulation of micronutrients or potentially toxic metals is influenced by the presence of organic material in seep sediments. The Guaymas case study demonstrates that hydrothermal seepage in sedimentary basins can create a significant nutrient flux and is an efficient means of recycling nutrients from organic matter at shallow burial depths. Hydrothermal nutrient fluxes could therefore have enhanced microbial activity in Earth’s history, in particular during time intervals when Earth’s oceans are thought to have been nutrient-depleted. Our data also highlight the role of organic material in enhancing metal mobilization and accumulation in otherwise metal-starved hydrothermal seeps.

## 1 Introduction

Hydrothermal systems are sites of pronounced thermochemical disequilibrium that play a significant role in nutrient supply to modern oceans (Gartman and Findlay, 2020). In particular, hydrothermal plumes are rich in metals that are vital for life (e.g., Fe, Co, Ni) and contribute to driving basin-scale biological productivity today (e.g., Schine et al., 2021). On the early Earth, hydrothermal vents may have played an even more significant role in sustaining life, before oxidative weathering started to supply bioessential metals to the oceans (Moore et al., 2017). In addition, hydrothermal fluids circulating through sediment packages may have provided a mechanism of remobilizing organic-bound nutrients such as ammonium (Stüeken et al., 2021a). In the anoxic oceans of the early Earth, remineralization of biomass in the water column was likely suppressed (Kipp et al., 2021), and so hydrothermal circulation may have partly filled this niche of nutrient recycling (Martin et al., 2024, 2025; Stüeken et al., 2021a,b).

While fossilized hydrothermal systems are a key focus for early life research, they prohibit quantitative estimates of recycling efficiency and nutrient fluxes because the timescales over which nutrient mobilization occurred are unconstrained. In this study, we focus on a modern analog, namely the Guaymas Basin in the Gulf of California, Mexico, where the interplay between active submarine magmatic-hydrothermal processes and large, organic-rich sedimentary systems can be examined.

**FIGURE 1:**
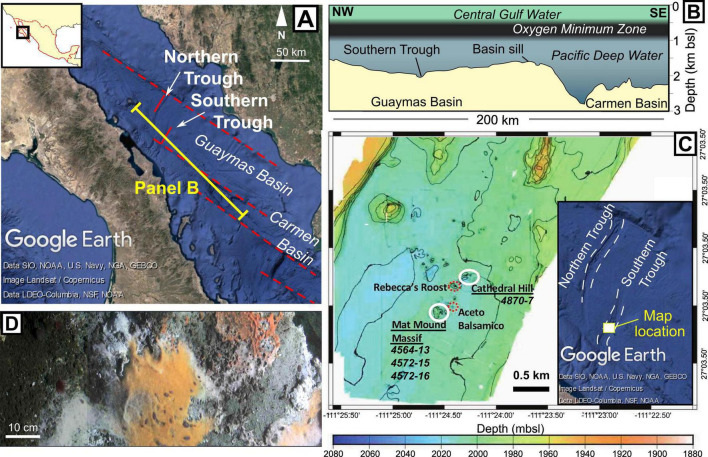
Study location. **(A)** The central Gulf of California with rift segments marked by red dashed lines. Positions of the Carmen and Guaymas Basins are indicated, as are the Northern and Southern Troughs within the Guaymas Basin. Satellite imagery and bathymetry are taken from Google Earth. **(B)** Sketched bathymetric profile of the Guaymas and Carmen Basins, based on a profile measured in Google Earth. The positions of the overlying water bodies are based on Bray (1988). **(C)** Bathymetric map of the central Southern Trough, modified from Teske et al. (2021), with inset bathymetry image from Google Earth showing location. Sampled areas (Cathedral Hill and Mat Mound Massif) are highlighted by white ellipses and respective core numbers are shown. Positions of other nearby hydrothermal seepage sites are indicated by red dashed ellipses. **(D)** *Beggiatoa* microbial mats on bare (gray) sediment, displaying three types of coloration that are found in the Guaymas Basin (white, orange and orange-red).

The Gulf of California (Figure 1) is an active continental rift system that initiated ca. 12–15 million years ago (Ma; Lizarralde et al., 2007), with long transfer faults separating a series of relatively narrow rift segments. The Guaymas Basin (Figure 1A) constitutes one of these segments, where seafloor spreading is thought to have initiated at roughly 6 Ma (Lizarralde et al., 2007). The basin is semi-enclosed, with highly productive surface waters underlain by an oxygen minimum zone (OMZ) between ca. 0.5 and 1 km water depth (Altabet et al., 1999). Between the base of the OMZ and the basin sill depth (c 1.5 km), oxic Pacific Deep Water enters and replenishes waters in the lower, enclosed part of the Guaymas Basin (Figure 1B; Bray, 1988).

As with other basins in the Gulf of California, the high productivity and rapid sedimentation in the Guaymas Basin have resulted in the deposition of >100 m thick, organic-rich diatomaceous sediments (Calvert, 1966; Lizarralde et al., 2007). Two ca. 150 m deep troughs in the basin, known as the Northern and Southern Troughs, represent the zones of active seafloor spreading (Figure 1; Teske et al., 2016). These active oceanic spreading centers are covered by sediment, meaning that igneous material is emplaced within the unconsolidated, organic-rich diatomite, rather than erupted as lava (DeMets, 1995; Lizarralde et al., 2007).

Igneous intrusions in the subsurface of the Guaymas Basin drive a variety of seafloor hydrothermal systems. Most of the hydrothermal systems identified to date occur in the Southern Trough (Figure 1C). However, the insulating properties of the overlying sedimentary package mean that igneous intrusions can also be found tens of kilometers off-axis, resulting in cold seeps and some hydrothermal vents at large distances from the axes of oceanic spreading (e.g., Ramírez et al., 2020; Teske et al., 2019).

Intrusion of magmatic material into organic-rich sediments in the Guaymas Basin stimulates hydrothermal systems that are rich in thermogenic alteration products including CO_2_, a variety of hydrocarbons (e.g., CH_4_; Dowell et al., 2016) and ammonium (NH_4_^+^; Von Damm et al., 1985). In the Southern Trough, hydrothermal systems create a complex topography of chimney and mound structures (Teske et al., 2016). Hydrothermal fluid flow also occurs via slower diffusive processes, with many hydrothermal seepage sites colonized by microbial mats (Figure 1D) and *Riftia* tubeworm colonies. The distinct white, yellow and orange microbial mats are dominated by large sulfur-oxidizing bacteria from the genus *Beggiatoa* (Jannasch et al., 1989; Teske et al., 2016). In addition, active microbial communities in surficial seep sediments include anaerobic methane-oxidizing archaea, diverse heterotrophs including fermenters and oil-degrading specialists, and ammonia-oxidizing bacteria and archaea that coexist in overlapping geochemical gradients on a scale of millimeters (Engelen et al., 2021).

The location and color of *Beggiatoa* mats reflect the hydrothermal seepage regime. These chemosynthetic bacteria store both elemental sulfur (S^0^) and nitrate (NO_3_^–^) intracellularly. The *Beggiatoa* use HS^–^ as an electron donor and NO_3_^–^ as an electron acceptor, reducing the latter to either N_2_ (denitrification) or NH_4_^+^ (dissimilatory nitrate reduction to ammonium, DNRA). The DNRA pathway generates more energy than denitrification per molecule of NO_3_^–^ and so is favored when HS^–^ is abundant. However, denitrification requires fewer electrons (five per NO_3_^–^ rather than eight for DNRA), rendering it favored under HS^–^-limited conditions and when the bacteria have to rely on stored electron donors (S^0^) within their cells (Schutte et al., 2018). White *Beggiatoa* mats are capable of DNRA and denitrification, whereas orange *Beggiatoa* mats are only capable of DNRA (Schutte et al., 2018). Thus, the “fried egg” appearance of many *Beggiatoa* mats (white at the fringes and orange in the middle) reflects the greater hydrothermal flow (and HS^–^ supply) supporting orange mats at the center of seeps, compared to lower flow sustaining white mats at the fringes.

To investigate the genesis and mobility of nutrient-rich fluids in the hydrothermal seep systems of the Guaymas basin that sustained the microbial mats, including *Beggiatoa*, we studied sediment and porewaters from active seeps. To ensure that the results are transferrable to studies of fossilized hydrothermal systems, we used established organic and inorganic geochemical proxies (e.g., Stüeken et al., 2021a,b), while also incorporating direct measurements of temperature and pore fluid composition. The geochemical proxy data were used to: (1) determine environmental (redox) conditions, (2) identify hydrothermal mobilization of nitrogen, phosphorus and nutrient metals in association with thermal alteration of organic-matter, and (3) draw inferences about the associated microbial processes. Temperature and porewater data were used to derive estimates of fluid seepage velocities and nutrient flux. The results were then combined with published observations from the basin to examine the efficacy of environmental redox proxies and to explore basin-scale implications of nutrient mobilization. By using this approach to study an active hydrothermal system, we provide new quantitative insights into sediment alteration and nutrient mobilization, which are evident in fossilized systems but extremely difficult to quantify directly. Thus, the results serve as a new reference point for studies of nutrient mobilization from hydrothermal seepage systems in the geological record.

## 2 Materials and methods

### 2.1 Sampling

**FIGURE 2:**
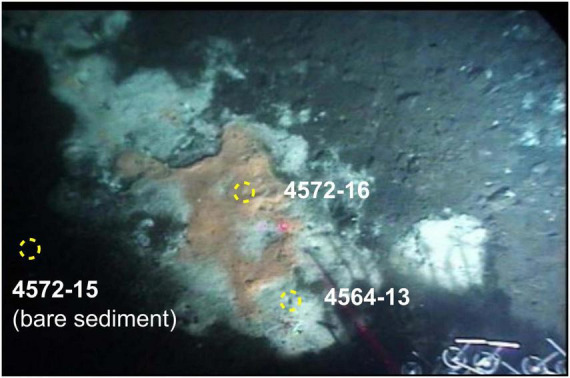
*Beggiatoa* microbial mat sampling site near Mat Mound Massif. The “fried egg” appearance of different *Beggiatoa* populations, with orange coloration at the center and white coloration at the periphery, corresponds to different intensities of hydrothermal upflow (McKay et al., 2012).

**FIGURE 3:**
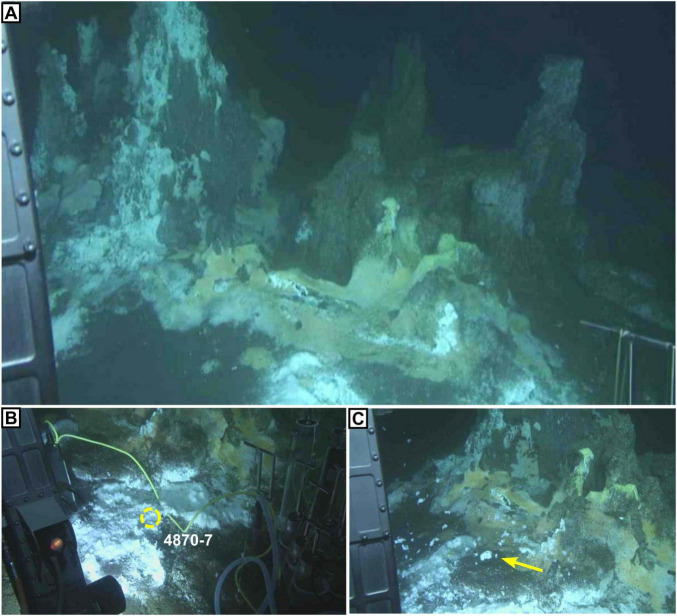
The “Witches’ Cauldron,” a highly active hydrocarbon- and sulfur-rich seep in the Cathedral Hill area with microbial mats visible on the surface. **(A)** Meter-scale towers formed of gray/brown baked oil-sediment concretions. White patches are sulfur flocs, which are only known from the most active seepage areas in the basin. Yellow patches are *Beggiatoa* mats. **(B)** Sample site for core 4870-7, brown-grey oily sediment at the base of the towers covered by a thin veneer of white sulfur flocs. **(C)** Disturbance of the sulfur flocs (yellow arrow) after temperature probe measurement (see also Supplementary Figure 1). Photos were taken during Alvin Dive 4870 on Dec 22, 2016, and are available on the Alvin framegrabber site (http://4dgeo.whoi.edu/alvin).

We analyzed shallow sediment cores from active hydrothermal seeps in the Guaymas Basin, covered with microbial mats (Teske et al., 2016). Four shallow (40 cm) push-cores from the Southern Trough of the Guaymas Basin were acquired by the DSV Alvin during RV Atlantis expeditions in 2009 and 2016 (Table 1). Three cores were taken from an active hydrothermal seepage site that was colonized by a *Beggiatoa* microbial mat, which displayed typical orange coloration at its center and white coloration at the periphery (Figure 2). Cores were taken from the center of the mat where orange *Beggiatoa* dominated (4572-15), from the periphery of the mat where white *Beggiatoa* dominated, (4564-13), and from bare sediment adjacent to the mat with no visible indicators of fluid seepage (4572-16). For comparison, an additional core (4870-7) was taken from a highly active seepage site rich in liquid hydrocarbons (oil), where the sediment surface was covered with white sulfur flocs; schlieren patterns marking the emergence of hot fluids were observed by the Alvin crew in this area (Figure 3; referred to informally as “the Witches’ Cauldron”). This type of seep forms meter-scale columns of sediment that is held together by a highly viscous mix of migrated hydrocarbons (bitumen) and some mineral precipitates including barite. The water-saturated samples were defrosted and centrifuged at 2000 rpm for 10 min to separate sediments from pore fluids. The supernatant was extracted using a syringe and passed through a 0.22 μm polyvinylidene fluoride (PVDF) filter. The pore fluids were stored in 50 mL falcon centrifuge tubes and amended with 100 μL of 2 *M* HCl, which was sufficient to give a slightly acidic (<6) pH and stabilize ammonium, before being stored in a freezer. The syringes, filters and centrifuge tubes were cleaned with deionized (DI) water before use. The remaining wet sediments were lyophilized over the course of 1 week, then stored in a freezer. As the sediment samples were not washed, corrections for porewater contributions of major seawater ions were determined gravimetrically from the lyophilized porewater masses.

**TABLE 1:** Details of the sediment push cores analysed in this study.

Core ID	Collection date	Location	Water depth	General description
4564-13	25/11/2009	27° 00.445 N/111° 24.530 W	2003 m	White *Beggiatoa* mats
4572-15	03/12/2009	27° 00.445 N/111° 24.530 W	2003 m	Brown sediment, no *Beggiatoa* mat
4572-16	03/12/2009	27° 00.449 N/111° 24.532 W	2003 m	Orange *Beggiatoa* mat
4870-7	20/12/2016	27° 00.710 N/ 111° 24.22 7 W	2007 m	Heavily oily sediment, with flocculous white sulfur precipitates

### 2.2 Stable isotopes

For organic carbon and nitrogen isotope analysis, roughly 0.5 g aliquots of dried sediment samples were mixed with 1 *M* HCl in glass centrifuge tubes and stirred with a glass rod. The tubes were then loosely capped and left to react in a fume hood at room temperature overnight. The next day, samples were centrifuged at 700 rpm, the supernatant was decanted and fresh 1 *M* HCl was mixed with the samples. After 4 hours, the samples were centrifuged and the supernatant decanted, before washing with DI water (stirring with a glass rod), centrifuging and decanting the supernatant. The DI water rinse was repeated a further two times, then the sample residues were dried in an oven at 70°C for 2–3 days. The dry samples were stored in glass scintillation vials. The glassware used for sample preparation and storage was pre-combusted at 500°C. Samples aliquots were weighed into 8 × 5 mm tin capsules and analyzed by flash combustion using an elemental analyzer (EA Isolink; Thermo Fisher) coupled via a Conflo IV to a MAT253 isotope ratio mass spectrometer (Thermo Fisher) at the University of St Andrews. Measurements were calibrated using the standards USGS-41a and USGS-40 and results are expressed in delta notation as (δ^15^N = [(^15^N/^14^N)_*sample*_/(^15^N/^14^N)_*standard*_ – 1] × 1000) versus air for nitrogen and (δ^13^C = [(^13^C/^12^C)_*sample*_/(^13^C/^12^C)_*standard*_ – 1] × 1000) versus the Vienna Peedee Belemnite (VPDB) standard for carbon. The USGS-62 (δ^13^C = −14.82 ± 0.10‰ [1σ], δ^15^N = 20.66 ± 0.24‰ [1σ], *n* = 12) and SDo-1 (δ^13^C = −30.35 ± 0.11‰ [1σ], δ^15^N = −0.56 ± 0.77‰ [1σ], *n* = 4) standards were used to test reproducibility, and the results agree well with published values (USGS-62: δ^13^C = −14.79 ± 0.04‰, δ^15^N = 20.17 ± 0.06‰, Coplen, 2019; SDo-1: δ^13^C = −30.0 ± 0.1‰, δ^15^N = −0.8 ± 0.30‰; Dennen et al., 2006).

### 2.3 Porewater analyses

Porewater NH_4_^+^ concentrations were determined following the colorimetric method of Cleaves et al. (2008). In short, 1 ml samples and standards were amended with 0.5 ml of phenol alcohol solution (1 ml liquified phenol with 90 ml of 100% ethanol, brought up to 100 ml using DI-water), followed by 0.5 ml of aqueous sodium nitroprusside (0.15 g of sodium nitroprusside dissolved in 200 ml of DI-water), and finally 1 ml of oxidizing solution (7.6 g of trisodium citrate and 0.4 g of sodium hydroxide in 500 ml of DI-water). The samples and standards were left to react for 60–80 min at room temperature, before analysis on a the Thermo Fisher Scientific Evolution Series 200 UV-Visible Spectrophotometer, at a wavelength of 640 nm.

Porewater δ^15^N was determined for a subset of samples using a microdiffusion method, which builds on that of Zhang et al. (2015). Ten milliliter samples containing 100 μM NH_4_^+^ were prepared from the porewater samples in 20 mL glass vials (focusing on samples with sufficient sample volume and NH_4_^+^ concentrations). To each sample, standard and blank, KCl was added to achieve a concentration of 1 M. The KCl was baked overnight at 500°C prior to use. One acid trap (a glass fiber filter with 10 μL of 2M H_2_SO_4_ sealed with water-proof Teflon tape; Zhang et al., 2015) was added to each of the vials along with a 5 mm magnetic stir bar. Next, ca. 100 mg MgO was added, and the vials were immediately crimp-capped with PTFE-lined butyl rubber septa. The solutions were placed in sand baths on magnetic stir plates at 70°C for 4 days, thus avoiding the need for a climate chamber and rotary shaker (e.g., Zhang et al., 2015). After incubation, the traps were removed, dipped in 1M HCl, followed by rinsing briefly in DI water. The acid traps were opened and placed in a freeze-drier. A fresh acidified glass fiber filter was also placed in the freeze-drier to test that no absorption of ammonia occurred during the drying process. The glass fiber filters were then extracted and placed in tin capsules for analysis using the same EA-IRMS system described above. Standards were prepared from 15 mL of 1 mM stock solutions of IAEA-N-1, IAEA-N-2, and USGS-25, which had been amended with 10 μl of 1 M HCl to prevent loss of volatile ammonia during storage. The USGS-25 and IAEA-N-2 standards were used for calibration, and IAEA-N-1 was used for quality control. The results for IAEA-N1 (δ^15^N = 0.2 ± 0.30‰ [1σ], *n* = 4) were in good agreement with published values (δ^15^N = 0.43 ± 0.14‰ [1σ]; Gonzalez and Choquette, 2023).

### 2.4 Trace element analysis

Major and trace element concentration data were acquired for aliquots of dried sediment by Australian Lab Services (ALS) in Dublin, Ireland. Following digestion in HNO_3_, HF, HClO_4_, and HCl, samples were analyzed via inductively coupled plasma mass spectroscopy (ICP-MS; MS-ME61r method). Reproducibility, based on duplicate analysis of a sample and the OREAS 920 standard, was better than 8% for the elements used in this study, with the exception of As, Ca and Sb, for which the reproducibility was better than 13%. Reproducibility for Ag with the OREAS 920 standard (44%; 0.15 ppm) was notably worse than the sample replicate (6%; 11.15 ppm), possibly due to low concentration. For three samples, the Ag concentration was also measured using inductively coupled plasma atomic emission spectroscopy (ICP-AES), following digestion in HF, HNO_3_, HClO_4_ and HCl (Ag – OG62 method). Reproducibility based on Ag replicate analysis with the OG62 method was 3.4%. We note that the reproducibility of certain elements was comparatively poor, possibly due to complex oil-bearing matrices. However, the relative trends discussed below are not impacted by the precision.

## 3 Results

### 3.1 Organically-associated metal enrichment

To examine the impact of migrated hydrocarbons on the delivery of nutrient metals to the seafloor, we used major and trace element data to examine element-specific enrichments associated with organic carbon in sediment. The trace element composition of the sediment is similar to upper continental crust, with alteration or “weathering” likely due to hydrothermal alteration (Supplementary Figures 1, 2). Thus, when discussing enrichment or depletion of chemical elements in sediment, we use Al-normalized element enrichment factors (EF) relative to an average upper continental crust composition (Rudnick and Gao, 2013) (X_*EF*_ = [X/Al]_*sample*_/[X/Al]_*UCC*_, where X is the element of interest and UCC refers to average upper continental crust.

**FIGURE 4:**
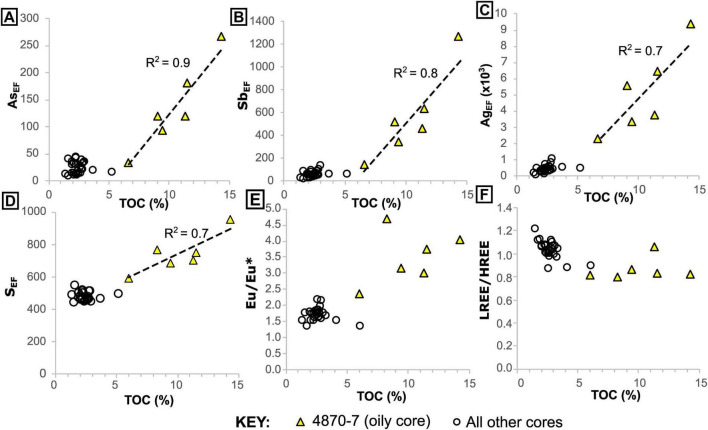
Elemental enrichment in the oily and non-oily cores, relative to total organic carbon. (**A–D)**, elemental enrichment factors **(E,F)** relative to UCC. **(E)** Europium anomalies and **(F)** light rare earth/heavy rare earth element ratio, relative to total organic carbon. Note the increased variability in organic carbon and elemental enrichment in the oily core samples.

**FIGURE 5:**
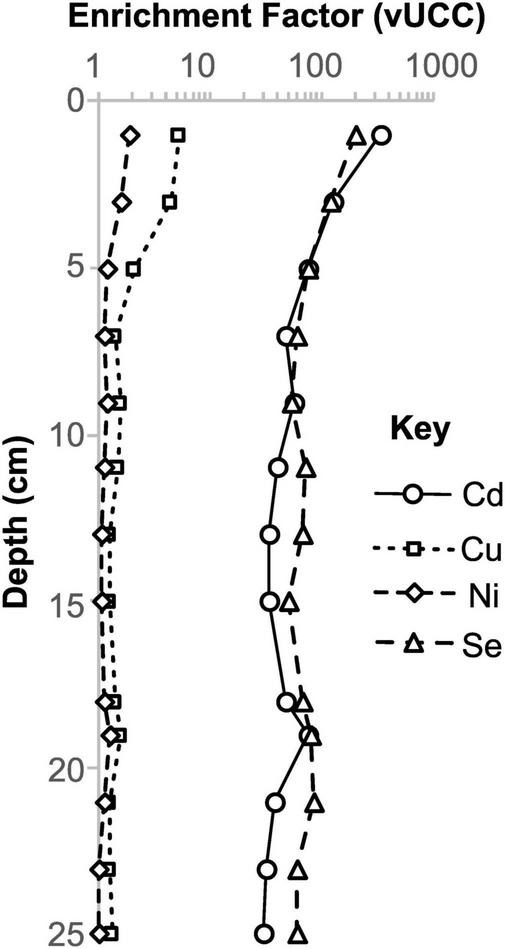
Selected elements that are enriched in shallow *Beggiatoa* mat sediments from core 4564-13.

In core 4870-7, we find several trace elements, including nutrient and toxic metals, which appear to have been mobilized by migrated hydrocarbons (oil) (Figure 4, Supplementary Figures 2, 4). In particular, Ag, As, S, and Sb have moderate to very high enrichment factors (up to Ag_*EF*_ = 9368) and show more covariance with total organic carbon (TOC) than the other cores (Figures 4A–D). Europium (Eu) anomalies (calculated as Eu_[SN]_/Eu_[SN]_* = 2 x Eu_[SN]_/Sm_[SN]_ + Gd_[SN]_, where SN indicates normalization to Post-Archean Australian Shale; Pourmand et al., 2012) are notably higher (up to 4.7) in the oily sediment samples, again showing a slight covariance with TOC (Figure 4E). The oily core has a lower overall rare earth element (REE) content with ΣREE (calculated as the sum of abundances of all REEs) between 25.0 and 63.3 ppm, in comparison to 98.2–177.6 ppm in the non-oily cores. This may be partly due to dilution by migrated hydrocarbons (Supplementary Figure 5), as this oil-rich core also contains slightly lower concentrations of detritus-associated elements like Al (see Supplementary data table in the BGS repository). It is also slightly enriched in heavy REEs (HREE/LREE calculated as [Pr/Yb]_*SN*_), compared to non-oily cores (Figure 4F). Barium is generally depleted in all the core samples, but shows slight enrichment toward seafloor in the oily core samples (Supplementary Figure 6). In the non-oily cores, the highest TOC values in sediments (which correspond to the seafloor microbial mat samples, rich in endogenous organic carbon) do not display covariance with the same elements (Figure 4). However, certain elements like Cd (maximum Cd_*EF*_ = 338) are associated with endogenous organic matter (Figure 5). Thus, we find that organic-rich samples are characterized by enrichment of certain metals and that samples rich in migrated (oil) and endogenous (microbial mat) organic matter have distinct signatures (Figures 4, 5, Supplementary Figure 4).

The accumulation of metals within sediment can also be influenced by environmental redox conditions. In anoxic/euxinic conditions, metallic elements in the water column can be bound within sulfide minerals or adsorbed onto sedimentary organic matter, which degrades more slowly than in oxic settings. Thus, it is important to understand whether some of the elemental enrichment observed could be associated with broader environmental deoxygenation, rather than hydrothermal fluids or microbial mat processes. Today, the bottom water of the Southern Trough is weakly oxic (Campbell and Gieskes, 1984). Redox indicators from shallow sediment (Supplementary Figures 1, 3) conform with this observation and indicate that broadly similar oxic conditions have prevailed in the water column above the sampling sites throughout deposition of the sampled intervals. Thus, it appears that the elemental enrichments observed are linked to microbial mat processes and deeply-sourced hydrocarbon-bearing hydrothermal fluids.

### 3.2 Sedimentary C, N and P data

**FIGURE 6:**
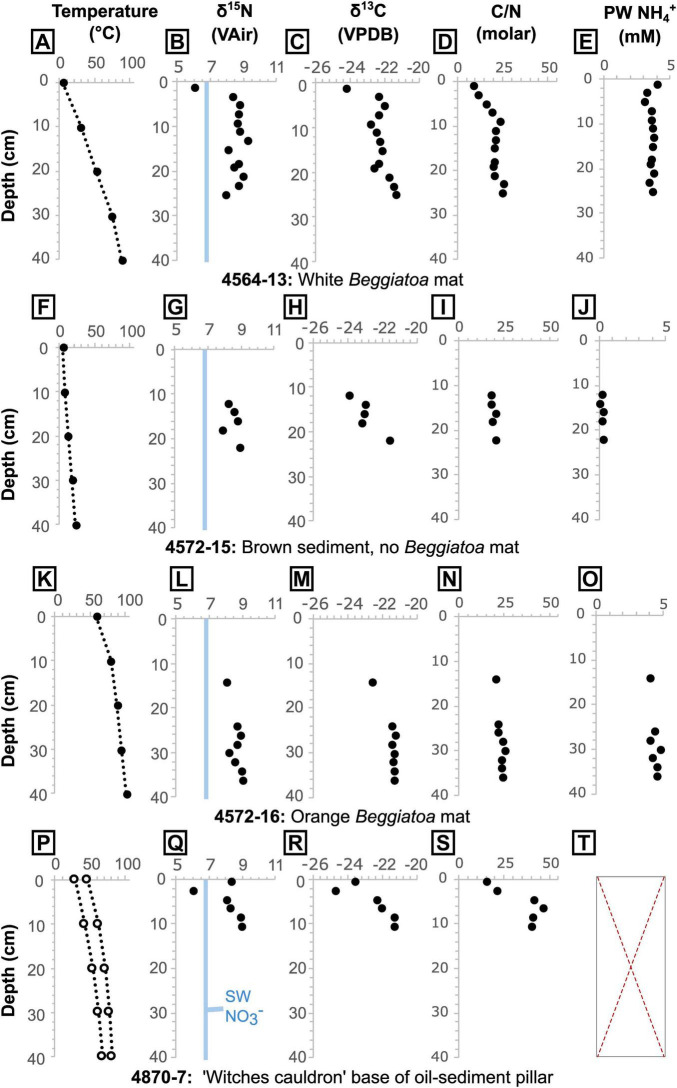
Core temperatures measured at 10 cm intervals via thermal probe, sediment δ^15^N, δ^13^C and C/N ratio, and porewater (PW) NH_4_^+^ concentration for cores **(A–D)** 4564-13**, (F–J)** 4572-15, **(K–O)** 4572-16, and **(P–T)** 4870-7. Core 4870-7 was taken between two temperatures profiles **(P)**. It was not possible to extract sufficient porewater from core 4870-7 for analysis **(T)**. An estimated δ^15^N value for the local seawater (SW) nitrate is shown in blue (see text for details). In the absence of direct measurements of local seawater nitrate (NO_3_^<suprm>–</suprm>^) δ^15^N, we take the approximate isotopic composition of Pacific Deep Water nitrate at 1500 m depth in the adjacent Carmen Basin (δ^15^N ≈ 6.8‰; Altabet et al., 1999), which is regarded as the source water for the deep Guaymas Basin (Campbell and Gieskes, 1984).

**FIGURE 7:**
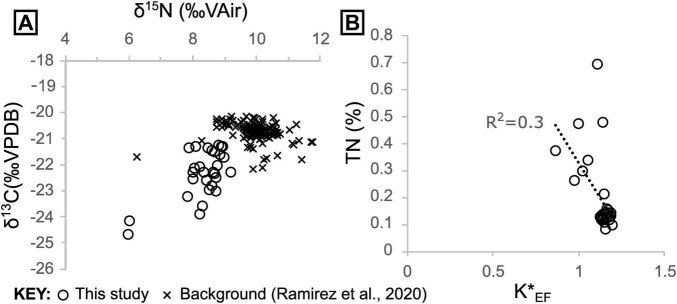
**(A)** Sediment δ^13^C and δ^15^N cross plot including data from this study and “background” samples taken outside of the Southern Trough from Ramírez et al. (2020). **(B)** Sedimentary nitrogen contents versus seawater-corrected K (denoted K*) enrichment factors.

We quantified organic C, along with total N and P, and performed stable isotope analyses of C and N to place our sediment samples into the context of hydrothermal vs. non-hydrothermal settings in Guaymas Basin. This allowed us to identify additional spatial trends that are associated with biological or abiotic chemical processes in seep environments. Sediment δ^13^C and δ^15^N data fall mostly within a narrow range (δ^13^C mean = −22.1 ± 0.8 [1σ], δ^15^N mean = 8.56 ± 0.4 [1σ], *n* = 30, excluding outliers) with two samples that are notable outliers, being depleted in both ^15^N (δ^15^N ≈ 6‰) and ^13^C (δ^13^C ≈−24.5‰) relative to other samples (Figures 6, 7). Our data are thus on average slightly lower in both δ^13^C and δ^15^N compared to the majority of sediment data that have been reported from outside of the hydrothermally active Southern Trough (δ^13^C mean = −20.7 ± 0.4 [1σ], δ^15^N mean = 10.0 ± 0.6 [1σ], *n* = 129; Ramírez et al., 2020; Figure 7).

**FIGURE 8:**
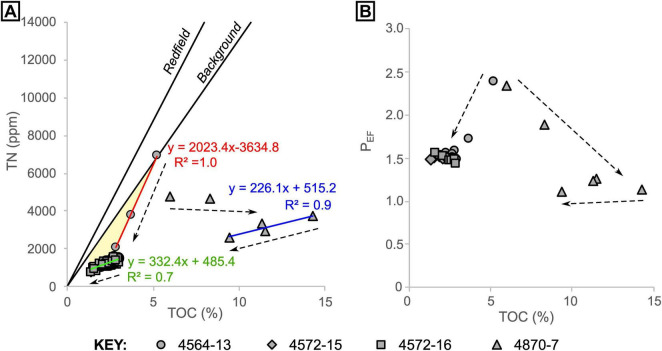
Total organic carbon versus **(A)** total nitrogen and **(B)** P enrichment factor for all samples, grouped by core. Arrows indicate general trends with increasing depth. The “Background” trendline in panel **(A)** was produced using pooled data from Ramírez et al. (2020) (*n* = 141, R^2^ = 0.9). The linear regressions in panel A are color coded to correspond to the equations and correlation coefficients. The yellow shaded area in panel **(A)** highlights depletion of nitrogen relative to carbon.

In cores 4564-13, 4572-15 and 4572-16, sediments below around 7 cm have a consistent C/N of 21 ± 2 [1σ]. For shallower samples (in core 4564-13), C/N ratios are lower but show an increase with depth, from a C/N of 9 to 21 (Figure 6). In the oily core (4870-7), values also increase with depth but are notably higher than in the oil-free cores, starting from 15 at 0.5 cm depth and stabilizing at around 42 from 4.5 cm depth downward (Figures 6, 8). The samples from core 4870-7 also follow a different trajectory to those from core 4564-13 (Figure 8). In core 4564-13, both carbon and nitrogen are lost with increasing depth. From the seafloor to around 7 cm depth, nitrogen is lost preferentially over carbon, and from 9 cm onward both are lost in more equal proportions, hence C/N is more stable (Figure 8A). In oily core 4870-7, nitrogen is lost progressively with depth, but TOC reaches a maximum (14.3%) at 6.5 cm depth. Similarly, phosphorus appears to be progressively depleted from surface to deeper sediments, from a maximum P_*EF*_ of 2.4 (corresponding to 1210 ppm P) to a P_*EF*_ of around 1.5 in the non-oily cores. The oily core (4870-7) also displays depletion from seafloor to depth, but the P_*EF*_ stabilizes at values of around 1.2 below 2.5 cm depth (Figure 8B).

Ammonium can substitute for K in mineral lattices, as the two share the same charge and similar ionic radii (Busigny and Bebout, 2013). However, K and N display a poor negative correlation (R^2^ = 0.33) (Figure 7B, Supplementary Figure 7), implying that most of the sedimentary N was introduced by biomass burial, rather than precipitation of ammonium-bearing hydrothermal mineral phases like K-feldspar or mica.

**FIGURE 9:**
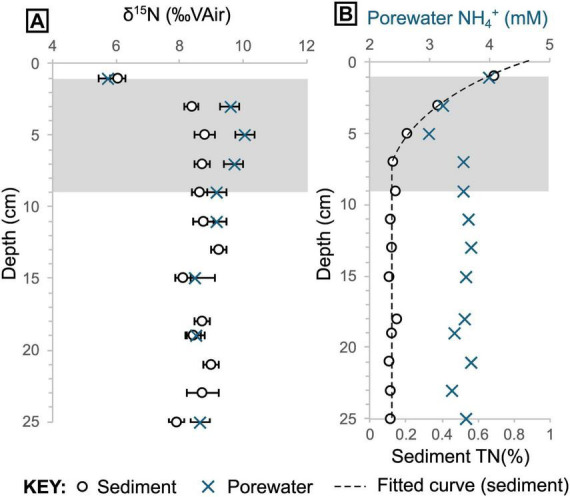
Total nitrogen and ammonium data from core 4564-13. **(A)** Sediment and corresponding porewater δ^15^N. **(B)** Porewater NH_4_^+^ (mM) and sedimentary nitrogen (%) concentrations plotted together. Shaded envelope marks the section of the core where sediment and porewater δ^15^N values begin to deviate notably in panel **(A)**. Note the elevated porewater δ^15^N in panel **(A)** and depleted porewater NH_4_^+^ concentration in panel B at 5 cm depth.

Porewater NH_4_^+^ concentrations show similar patterns throughout each core analyzed, but the concentrations vary between cores, with samples from hotter areas containing higher NH_4_^+^ concentrations (4572-15 = 0.3 mM ± 0.2 [1σ], T_*max*_ = 14°C; 4564-13 = 3.5 mM ± 0.2 [1σ], T_*max*_ = 63°C; 4572-16 = 4.4 mM ± 0.3 [1σ], T_*max*_ = 98°C). In core 4564-13, the porewater NH_4_^+^ concentrations are relatively constant from the base up to 9 cm with a mean of 3.58 mM (± 0.11 1σ) before dropping to 3.0 mM at 7 cm depth (Figure 6). The concentrations then increase again to 4.0 mM at the top. Porewater δ^15^N_*NH*4+_ data for the two hotter cores are very similar across the same depths (pooled δ^15^N_*NH*4+_ mean = 8.6 ± 0.1 [1σ]; Figure 5, Supplementary Figure 8), though no samples were available for depths shallower than 9 cm in core 4572-16. For the shallower microbial mat samples (in core 4564-13), δ^15^N_*NH*4+_ reaches a maximum value of 10.0‰ at 5 cm depth, before dropping to a value of 5.7‰ at 1 cm depth (Figures 6, 9). Porewater δ^15^N_*NH*4+_ is generally within error of sedimentary δ^15^N values, except between roughly 9 cm and 3 cm depth in core 4564-13, where porewater δ^15^N_*NH*4+_ is up to 1.3‰ higher (Figure 9).

### 3.3 Quantification of flow velocities and nitrogen fluxes

We used two approaches to explore the local and basin-scale implications of our analytical results. First, we calculated the ammonium seepage fluxes at three core sites using temperature and porewater data. We then used the sediment data to constrain the contribution of shallowly derived (< 40 cm depth) sedimentary nitrogen to the total basin hydrothermal flux. Details of the upscaling approaches used are presented in Supplementary File S2.

To explore differences in flow regime across one of the seepage sites, hydrothermal velocities were estimated at 20 cm depth in three of the cores, using the approach of Bredehoeft and Papaopulos (1965) for quantifying heat and fluid transport. Ammonium fluxes were then calculated using corresponding porewater data. The calculated velocities indicate both discharge (−8.8 to −2.9 × 10^–7^ m s^–1^) and recharge (4.4 × 10^–7^ m s^–1^; Table 2). The corresponding (absolute) hydrothermal ammonium fluxes at the seepage site are 0.13–3.68 μmol m^–2^s^–1^ (Table 2). The maximum seepage velocity (from the central, orange part of the mat; Table 2) is higher than the maximum velocity calculated using the same approach at an off-axis vent site (maximum of ca.8 × 10^–9^ m s^–1^, assuming porosity of 80%; Neumann et al., 2023), due to higher temperature and heat flow.

**TABLE 2:** Calculated flow velocities for cores at 20 cm depth, with corresponding porewater ammonium fluxes calculated using thermal data and average porewater ammonium concentration from the two closest samples in each core. Core 4870-7 is omitted due to a lack of extractable pore fluid. The sedimentary ammonium loss was calculated using sediment N data from the top of 4564-13 (it was not possible to apply this calculation to the other cores due to lack of samples). Negative values indicate upward migration.

Core ID	Calculated flow velocity at 20 cm depth (m s^1^) based on thermal data	Porewater ammonium flux (μmol m^2^s^1^) based on thermal and pore fluid data	Sedimentary ammonium loss (μmol m^2^s^1^)
4564-13	−2.9 × 10^–7^	−1.04	−0.00433 to −0.0135
4572-15	4.4 × 10^–7^	0.13	–
4572-16	−8.8 × 10^–7^	−3.68	–

The flow velocities calculated above are useful for exploring local variability in flow regime, but also highlight that a more complex fluid flow model would be required to upscale and estimate the corresponding hydrothermal ammonium fluxes from the whole Trough. Thus, in our second approach we examined the core sediments as a time-integrated (i.e., long-term) record of nitrogen loss. A piecewise function was fitted to the sedimentary nitrogen abundance data from 4564-13 (Figure 9B, Supplementary File S2). The function follows an exponential curve of the form y = y_0_ ⋅ a*^x^* (where y = nitrogen content, y_0_ = nitrogen content at seafloor, a = loss coefficient, and x = depth) from seafloor to ca. 7 cm depth. We assume that the remaining nitrogen is resistant to hydrothermal remobilization in the remaining interval. The curve gives an integrated nitrogen loss of around 54% within the top ∼7 cm of sediment (Supplementary File S2). If we assume a sedimentation rate of roughly of 0.08–0.25 cm/yr (Calvert, 1966; DeMaster, 1981; Geilert et al., 2018), this would correspond to an ammonium loss flux of 4.33–13.52 nmol m^–2^s^–1^ from the shallow sediments in the seepage area (Table 2).

## 4 Discussion

### 4.1 Hydrothermal mobility of biologically-active metals and organic complexation

Hydrothermal systems at oceanic spreading centers can supply metallic nutrients and drive productivity at a regional scale (Schine et al., 2021). Given the widespread evidence of hydrothermal activity, it might be expected that bio-essential metals like Cu, Fe, Ni and Zn are relatively abundant in the hot sediments of the Southern Trough. However, Guaymas Basin hydrothermal fluids are strongly depleted in ore-forming metals overall, unlike those in sediment-starved oceanic spreading centers where black-smoker type vents typically form (Von Damm et al., 1985). This relative metal depletion is due to the presence of a thick sediment package, which is related to high productivity in surface waters (Calvert, 1966; Lizarralde et al., 2007). Water-sediment interactions, including the dissolution of carbonate and liberation of sedimentary ammonium, are thought to raise the pH and alkalinity of hydrothermal solutions in the Guaymas Basin, resulting in precipitation of metal sulfides deep below the seafloor (Seewald et al., 2024; Von Damm et al., 1985). Slight metal enrichment in sediment (e.g., Ag, Cd) has been linked to surface water export productivity (Chang et al., 2015). However, for the samples analyzed in this study, we find moderate to extreme enrichment of metals (e.g., Ag, As, Sb, Zn; Figure 4, Supplementary Figure 4) in association with migrated hydrocarbons in the oily core (4870-7). The local enrichment of Ag at this site (up to Ag_*EF*_ = 9368 at 4.5 cm depth, corresponding to 120 ppm) is equivalent to high grade silver ore; higher than the average concentrations found in Volcanogenic Massive Sulfide (VMS, ca. 33 ppm) and sedimentary exhalative (SEDEX, ca. 46 ppm) silver deposits (Graybeal and Vikre, 2010). We interpret this as the result of organo-metallic complexation, which facilitates the transport of metals in fluids when they may otherwise precipitate or remain trapped in biomass at depth. Europium anomalies in the same samples (Figure 4E) suggest that oil migration is indeed linked to hydrothermal fluids, which may have supplied the metals from deeper in the subsurface. Furthermore, high HREE/LREE (Figure 4F) suggests that HREE are also incorporated preferentially, or retained more easily, in the liquid organic phase. Thus organometallic complexation appears to be a means of overcoming chemical barriers to hydrothermal transport of metals in this sediment-rich oceanic spreading system.

Once metal-bearing hydrothermal fluids reach the seafloor, microbial processes could provide additional mechanisms for concentrating transported metals in sediment. Sulfate-reducing metabolisms are found within meso- and thermophile microbial communities in the Guaymas Basin (Teske, 2020). Enrichment of S in the oily core (4870-7) is partly a reflection of the vigorous flow regime in the area, which is evidenced by the presence of elemental sulfur flocs on the seafloor. The abundance of migrated hydrocarbons in the sediment at this site could also have stimulated the activity of heterotrophic sulfate-reducing microorganisms (e.g., bacterial-fungal associations; Edgcomb et al., 2022), leading to local enrichment of S in the form of sulfide. Thus, elements with an affinity for sulfur (chalcophiles) could be bound within biogenic S minerals like pyrite deeper in the sediment. The abundant bitumen in the cohesive sediment columns at Witches’ Cauldron is a further indicator of biological and/or thermal degradation of lighter hydrocarbons, which may further concentrate trace metals in the residue at this site. Unfortunately, the migration of hydrocarbons and hydrothermal fluids through the sediment makes it difficult to confidently resolve the relative contributions of sulfide- and organically-complexed chalcophile elements in the oily sediment samples.

Certain elements (e.g., Cd, Cu, Ni, Se) are notably enriched within shallow *Beggiatoa* mat (non-oily) samples (Figure 5), despite oxic conditions in the overlying water column (Supplementary Figure 1; Campbell and Gieskes, 1984), suggesting a passive or possibly active biological control on their accumulation. The oxidation of HS^–^ by *Beggiatoa* has been shown to decrease pH locally within laboratory-maintained mats (Schutte et al., 2018), a likely consequence of sulfur oxidation. In contrast, sulfide accumulates in the deeper mat where oxygen does not penetrate. It is possible that this sulfide accumulation, or other metabolic processes, are responsible for the accumulation of certain elements within the mats, perhaps in combination with organo-metallic complexation. Elements enriched in mats at shallow depths appear to be lost during burial (Figure 3), along with more labile organic matter. The shallow mat sediments are therefore dynamic reservoirs for these additional metals.

Many of the elements that are enriched in oily samples (e.g., Zn, Supplementary data table in BGS repository and Supplementary Figure 4) are important nutrients for the biosphere, while others (e.g., Ag, As, Cd) can impair cellular function. Microbial communities in extreme environments are adapted to cope with toxic metals and high metal concentrations. This is often achieved through efficient efflux mechanisms, as with organisms like *Thermococcus* and *Pyrococcus* (Bini, 2010), which have been isolated from mat-related sediments in the Guaymas Basin (Teske et al., 2009). *Beggiatoa* mats are found in certain areas at Witches’ Cauldron; however they were not present at the 4870-7 coring site (Figure 3), despite an abundance of sulfur-rich hydrothermal fluid. This may relate to locally elevated temperatures at this site that, combined with strongly reducing conditions, instead favor the development of microbially produced filamentous sulfur flocs by organisms like *Arcobacter spp.* (Taylor and Wirsen, 1997). The enrichment of potentially toxic metals like Ag may be an additional control on the microbial ecology at the Witches’ Cauldron and similar sites in the Guaymas Basin. Thus, our findings highlight that metals transported by thermogenic hydrocarbons and/or concentrated within mats could be exerting a control on microbial community composition at the seafloor, by providing both nutrients and potentially toxic metals.

### 4.2 Sedimentary nitrogen mobilization

The interaction between hydrothermal fluid seepage and organic matter motivates further investigations into the behavior of important organic-bound nutrients (e.g., nitrogen) in the system.

The shallowest (microbial mat) sample from core 4564-13 has a N/C ratio (N/C [g/g = 0.133) that is similar to background (non-hydrothermal) sediments (N/C [g/g] ≈ 0.131; Ramírez et al., 2020). These values are slightly lower than the Redfield N/C ratio for fresh biomass (ca. 0.18 [g/g]; Redfield, 1958), as the sedimentary organic matter has likely experienced a small amount of alteration in the water column (e.g., in the OMZ) or at seafloor (Figure 8A). However, N/C ratios abruptly become even lower further below seafloor. The trend of decreasing N/C from seafloor to ∼10 cm depth indicates that nitrogen is then lost preferentially over carbon. After this point, carbon is lost slightly more than nitrogen with increasing depth in all of the non-oily cores (Figure 8A). We interpret this loss of nitrogen to be the result of hydrothermal remobilization of ammonium from organic matter, in agreement with low N/C ratios elsewhere in the Southern Trough (De La Lanza-Espino and Soto, 1999). In core 4870-7, the conspicuous enrichment of C (maximum TOC = 14.3% at 4.5 cm depth) is interpreted to reflect the addition of migrated hydrocarbons.

In the non-oily cores, P_*EF*_ drops rapidly over the same depth interval as nitrogen, before stabilizing at P_*EF*_ ≈ 1.5 (Figure 8B). Thus, it appears that P, another biologically-limiting nutrient, is also being lost from sedimentary organic material as a result of hydrothermal alteration. In the oily core, P_*EF*_ drops with depth but stabilizes at approximately crustal values (P_*EF*_ ≈ 1). The enhanced P depletion in the oily core could be associated with enhanced heterotrophic microbial activity and sulfate reduction, which would result in additional organic-P remineralization. The results highlight that hydrothermal fluids can directly (via thermal alteration) and indirectly (via stimulation of heterotrophic activity) facilitate recycling of essential nutrients from buried biomass.

### 4.3 Quantifying nitrogen mobilization

The calculated fluid flow velocities at three of the core sites are highly variable (Table 2). This is because hydrothermal circulation creates a complex field of fluid flow directions and magnitudes in the shallow sediments of the Southern Trough. The corresponding (absolute) hydrothermal ammonium fluxes at the seepage site (0.13–3.68 μmol m^–2^s^–1^; Table 2) are ca. three orders of magnitude higher than the estimated nitrogen input from sedimentation (3.73–11.65 nmol m^–2^s^–1^; Supplementary File S2). Since hydrothermal discharge zones (vents and seeps marked by *Beggiatoa* mats) occupy a relatively small proportion of the seafloor in the Southern Trough, the loss via hydrothermal discharge over a small total area more closely balances the supply from widespread sedimentation across the whole of the Trough.

We expect that the estimated ammonium flux from shallow sediment (4.33–13.52 nmol m^–2^s^–1^) will be more representative across a larger area of the Southern Trough. Although the lack of shallow samples from the other (non-oily) cores prevents direct comparison across sites, the C and N data consistently indicate nitrogen losses of strikingly similar magnitude within the top <10 cm of sediment (Figures 6, 8), despite differences in temperature and flow velocity at the time of coring. This may reflect the temporal variability in the hydrothermal flow regime, with short-term changes in flow pathways resulting in an overall similar magnitude of nitrogen loss over an area. Hydrothermal flow pathways in the Southern Trough are known to be highly transitory, with seeps and associated thermal anomalies that can disappear within less than a year (Teske et al., 2016). The results could therefore be applicable across large areas of the basin with similar heat flow (Williams et al., 1979).

The ammonium fluxes calculated from temperature and porewater data in cores 4564-13 and 4572-16 (0.13–3.68 μmol m^2^s1; Table 2) exceed the rate at which nitrogen is being lost from the sediment (i.e., the sediment loss flux of 4.33–13.52 nmol m^–2^s^–1^; Table 2). Most likely, this result reflects the exogenous origin of the majority of the porewater ammonium, which has migrated with hydrothermal fluids into the sediment from elsewhere (e.g., from deeper sediments).

By upscaling the loss of nitrogen that we observe from shallow sediment in the Southern Trough, we can now explore the contribution of shallowly-buried biomass to the total basin hydrothermal ammonium flux. More than half (ca. 54%) of the sedimentary nitrogen is lost rapidly in the surface (<10 cm) sediment at the non-oily seepage site (Figure 9B). However, to place this result in context, we also need to account for ammonium released from deeper sediment, which likely forms a larger component of the fluids released from hotter, channelized vent systems in the Basin. Campbell and Gieskes (1984) proposed a total hydrothermal fluid flux of around 10–12 m^3^ s^–1^ into the Guaymas Basin, which includes fluids from seepage sites and channelized vents. This corresponds to a total ammonium flux of around 156.0–187.2 mol s^–1^ using their hydrothermal fluid endmember data (15.6 mM NH_4_^+^), which was gathered from a hotter and faster-flowing system in the basin (Von Damm et al., 1985; Supplementary File S2). Extrapolating our shallow nitrogen loss estimate across the hydrothermally active Southern Trough (area ca. 100 km^2^) and Northern Trough (area ca. 200 km^2^) yields a total flux of 1.3–4.1 mol s^–1^. This flux constitutes ca. 2% of the total basin hydrothermal ammonium flux. The magnitude of nitrogen loss is remarkably high in the top ∼10 cm of seep sediment (Figure 9B), but this result highlights that the majority of hydrothermal ammonium in the Guaymas Basin (98% in this estimate) is likely sourced from deeper sediments, via high-temperature vents and possibly other off-axis seeps that are unaccounted for in our estimate.

### 4.4 Implications for microbial nitrogen utilization by seep communities

**FIGURE 10:**
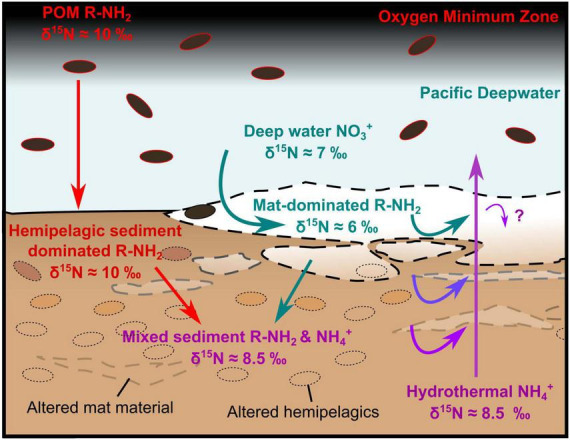
Sketch illustration of nitrogen cycling around a hydrothermal seep in the Guaymas Basin’s Southern Trough. Beggiatoa-dominated microbial mats assimilate Pacific Deepwater nitrate, while the surrounding sediment surface is dominated by hemipelagic sediment (diatomite). The lower parts of the mat are progressively degraded, liberating ammonium, which is mobilized by hydrothermal fluids that also carry ammonium from depth. The deep sediment represents a mix of more recalcitrant mat-derived and hemipelagic material, which has a consistent δ^15^N laterally. The question mark “?” highlights uncertainty as to whether some of the hydrothermal NH_4_^+^ is utilized by mat organisms. Particulate organic matter is abbreviated as POM.

Having established the presence of hydrothermal ammonium seepage, we first explore the effects of this flux locally on microbial communities, using isotopic data, before discussing broader basin-scale effects on nutrient budgets. Sedimentary δ^13^C and δ^15^N are generally ca. 1.4‰ lower than the pelagically-dominated background sediments outside of the Southern Trough (Figure 7). There, δ^15^N is on average relatively high (9.97‰, ± 0.64 [1σ]; Ramírez et al., 2020) compared to the global marine average of 5–6‰, due to microbial denitrification in the overlying OMZ (at ca. 500–1000 m water depth; Bray, 1988; White et al., 2013). The light δ^15^N (ca. 6‰) of the seafloor microbial mat sample in core 4564-13 points to assimilation of Pacific Deep Water-derived nitrate by the white *Beggiatoa* community residing on the seafloor (Figures 6, 10). The overall absence of a 6‰ sedimentary δ^15^N signature in non-hydrothermal sediments outside of the Southern Trough (Ramírez et al., 2020) may reflect an absence of nitrate-assimilating microbial mats in those settings. Below the mat-impacted surface layer, the increasing δ^15^N isotopic values reflect hydrothermal loss of isotopically light nitrogen from more labile organic material, leaving the more recalcitrant mat-derived and pelagic organic material (Figure 10). The isotopic signature of deeper core samples is fairly consistent and still lighter than background (non-hydrothermal) sediments (Figure 7). Thus, it appears that nitrate was also available for microbial assimilation during deposition of the older sediments, which implies the presence of a broadly oxic water column during deposition of the cored intervals, and complements the redox indicator data (Supplementary File S1). In contrast to 4564-13, core 4870-7 (oily core) has a heavy δ^15^N isotopic signature at the seafloor and in deeper samples, but light isotopic values at ca. 2.5 cm depth (Figure 6). No *Beggiatoa* mat was observed at the seafloor in this location at the time of sampling, possibly due to high temperature pulses that exceed the limited thermal tolerance of *Beggiatoa* (McKay et al., 2012); the site was instead dominated by voluminous sulfur flocs. The δ^13^C and δ^15^N isotopic signatures of all cores are lighter than sedimentary background values from outside of the Southern Trough (Figures 7, 9). Thus, it may be possible to distinguish nitrate assimilation by seep biota in the geological record, if the isotopically heavier background sediments are also preserved.

Localized isotopic fractionation and concurrent NH_4_^+^ depletion in the white *Beggiatoa* mat sediments in 4564-13 could indicate some microbial utilization of porewater NH_4_^+^ (Figure 9). However, the calculated isotopic fractionation (ca. 10‰; Supplementary Figure 9) is lower than measured values associated with other NH_4_^+^- consuming metabolic processes (e.g., anammox, 23.5–29.1‰; Brunner et al., 2013) that might be expected. The overall muted isotopic signature of δ^15^N enrichment in sediments likely reflects a mix of competing metabolic processes and possibly nitrogen substrates in the shallow mat sediments. The results are striking in that they indicate the dominant source of organically-bound nitrogen in the *Beggiatoa* mat communities is seawater-derived δ^15^N-fractionated nitrate, rather than the abundant, more ^15^N-enriched hydrothermal ammonium percolating from below. This result is consistent with uptake of seawater nitrate into membrane-lined vacuoles that dominate the cytoplasmatic biovolume of Guaymas *Beggiatoa* filaments (Jannasch et al., 1989), resulting in intracellular nitrate accumulation by approximately three orders of magnitude (Buckley et al., 2019).

### 4.5 Implications for basin-scale ammonium utilization

Having placed new quantitative constraints on the magnitude and timeframe of nitrogen loss from shallow sediment, we can incorporate published records from this well-studied basin to explore possible basin-scale implications for microbial productivity. We acknowledge limitations with regard to the spatial extent of our sample set and highlight that the ammonium loss from shallow sediment will vary across the hydrothermally active Southern and Northern Troughs (as does heat flow; Neumann et al., 2023; Williams et al., 1979). Often, geochemical studies of fossilized systems are faced with similar limitations due to lack of preservation or geological exposure. As discussed above, temporal variability in the local seepage regime has resulted in a strikingly similar (overall) magnitude of nitrogen loss across the core sites, despite differences in temperatures and hydrothermal flow velocities at the time of coring. Thus, upscaling these nitrogen loss results across a larger area could still provide a new and useful insight into the potential biospheric impact of shallow hydrothermal nitrogen recycling in the basin.

Extrapolating the observed shallow sedimentary nitrogen loss across the hydrothermally active Southern and Northern Troughs, the liberated nitrogen could sustain up to around 10^–7^ kg of biomass (as dry carbon) each year (Supplementary File S2), assuming a molar C:N ratio of 106:16 for fresh biomass. As an exercise, if we assume steady state, complete vertical mixing and treat the Guaymas Basin as closed, the remobilized nitrogen could sustain additional productivity of around 19–61 mmol C m^–2^yr^–1^ (Supplementary File S2) in the basin. These values are equivalent to roughly 0.4%–1.3% of the typical biological export production in the basin (ca. 4.8 mol C m^–2^yr^–1^; White et al., 2013). Using the total hydrothermal flux estimate from Campbell and Gieskes (1984), which includes high-temperature vents in addition to seeps, these values increase to 48%–58% (Supplementary File S2). For context, N_2_ fixation contributes 0.4%–44.2% of the measured export productivity in the neighboring areas of the Gulf of California (White et al., 2013). We emphasize that the hydrothermal nitrogen flux may not reach the photic zone of the Guaymas Basin directly, because the basin exhibits seasonally-variable stratification at several depth horizons (Bray, 1988). The basin is semi-confined and although hydrothermal plume signatures can be detected in the water column at sub-sill depths (ca. > 1500 m; Lam, 2004), strong tidal currents (Lonsdale and Becker, 1985) mean that this hydrothermal signature is lost abruptly due to dilution by Pacific Deepwater above the basin sill depth (Campbell and Gieskes, 1984; Lam, 2004), and much of the hydrothermally-derived nitrogen is likely exported out into the Gulf. However, the Guaymas Basin exhibits net outflow at shallow (<250 m) depths and net inflow at greater depths (Bray, 1988), which facilitates seasonally enhanced upwelling and may aid the transfer of hydrothermally derived nitrogen to shallower depths, i.e., into the OMZ and possibly photic zone, where most productivity takes place. Our findings thus provide a first-order assessment of the impact that hydrothermal nutrient remobilization may have on biological productivity, in particular in ancient settings with a lower nitrate inventory and perhaps lower rates of biological N_2_ fixation.

### 4.6 Conclusion and outlook

We deployed techniques that are commonly used to understand nutrient cycling in the rock record, in combination with porewater analysis and published observations from the hydrothermally active Guaymas Basin. In doing so, we have highlighted some of the dynamics within this system that would be obscured in the rock record.

The mobility and accumulation of trace metals appears to be enhanced through association with migrated hydrocarbons and microbial mats at the study sites, despite being an overall metal-poor system. This could occur directly, through processes like organometallic complexation with migrated hydrocarbons, or indirectly, via metabolic processes like heterotrophy and sulfide precipitation. Several metals are concentrated within hydrocarbon-rich seeps, including ore-grade concentrations of Ag. Seafloor microbial mats are enriched in metals like Cd, but the breakdown of buried mat material via hydrothermal and biological processes means that the shallow sediments function as dynamic reservoirs for those elements. Thus, the enrichment patterns of nutrient or potentially toxic metals highlighted here could be exerting a control on microbial community composition (Edgcomb et al., 2004).

We have also shown that breakdown of organic material via hydrothermal activity liberates important nutrients like phosphorus and nitrogen from sediment, which could be contributing to enhanced productivity higher in the water column. The results show a contrast in terms of isotopic composition and mobility of the endogenous (mat-derived) and exogenous (pelagic-dominated) nitrogen fractions in the sediment. Often it is assumed that the δ^15^N composition of sediment is representative of the parent organic material is (e.g., Boudou et al., 2008). However, in this study, the mix of labile and δ^15^N-depleted mat material at the sediment surface and more recalcitrant and mature δ^15^N-enriched particulate material in the underlying sediments at the microbial mat site results in an isotopic shift during nitrogen loss, possibly also linked to microbial activity.

Remobilization of nitrogen from sediments due to fluid flow has been invoked to explain depleted N/C ratios in several geological studies, which have in-turn been used to make inferences about nutrient cycling in deep time (e.g., Martin et al., 2024, 2025; Stüeken et al., 2023; Stüeken et al., 2021a,b). Here, we have observed active loss of nitrogen from sediment in a hydrothermal seep. The data from core 4564-13 show that vigorous fluid seepage in areas of high heat flow (e.g., a young oceanic spreading axis) can liberate more than half the accumulated sedimentary nitrogen within as little as 27–83 yr (Supplementary Figure 2). This effect appears less pronounced in off-axis areas, where injected magmatic bodies are of lower volume and occur more sporadically (e.g., Ringvent; Ramírez et al., 2020). Together with high-temperature vents, hydrothermal seepage in sedimentary basins thus has the potential to act as an important mechanism for recycling nutrients. Our findings also support the notion that in ancient oceans, where the overall nutrient inventory was perhaps lower than it is today and oxidative remineralization of organic matter in the water column was suppressed (Kipp et al., 2021), hydrothermal circulation through sediment packages could have boosted microbial productivity.

## Data Availability

All data generated in this project are available from the National Geoscience Data Centre of the British Geological Survey under: https://doi.org/10.5285/53da3dd9-e831-4c4e-8879-c8e8a014ed4d. Supplementary File S2, containing code generated in this study, is available in a Zenodo repository with the identifier: https://doi.org/10.5281/zenodo.15782744.
