# Supplementary material for: Quantifying hydrothermal ammonium mobilization from sediment and implications for the marine biosphere: a case study from the Guaymas Basin, Gulf of California
Source: Front Microbiol. 2025 Jul 16;16:1523696. doi: 10.3389/fmicb.2025.1523696 (PMC12309410; doi:10.3389/fmicb.2025.1523696)
Supplement: Supplementary file 1 [file Supplementary_file_1.docx]

**Supplementary S1: additional text and figures for** ‘**Quantifying hydrothermal ammonium mobilization from sediment, and implications for the marine biosphere: A case study from the Guaymas Basin, Gulf of California’**

Nathan Rochelle-Bates^1^*, Annabel Long^1^, Graeme MacGilchrist^1^, Andreas Teske^2^, Eva E. Stüeken^1^

1. University of St Andrews, School of Earth & Environmental Sciences, Bute Building, Quen’s Terrace, St Andrews, Fife, KY16 9TS, United Kingdom

2. University of North Carolina at Chapel Hill, Department of Marine Sciences, Chapel Hill, NC 27599-3300, United States of America

* corresponding author ([nrb1@st-andrews.ac.uk](mailto:nrb1@st-andrews.ac.uk))

**Supplementary text**

**S1.1 Sediment provenance**

To identify hydrothermal signatures in sediment, it is important to have an understanding of the original sediment composition. Thus, we first used a simple mixing model with two element ratios to determine the sediment provenance (Ni/Yb and Sc/Zr; Ptáček et al. 2020) (Fig. S2). The majority of sediments roughly plot on the mixing curve between modern mafic and felsic endmembers (Ptáček et al., 2020), close to average upper continental crust (Rudnick and Gao, 2013). Sediments also fall within a very narrow range of Sc/Zr ratios (average = 0.0934 ± 0.0033 [1σ]), but some deviate from the mixing curve along the Ni/Yb axis (Fig. S2A). Nickel, like several other transition metals, is useful for distinguishing komatiitic sediment components, but can also be enriched within hydrothermal systems (Ptáček et al., 2020). Thus, hydrothermal alteration is likely the cause of the enrichment in these sediments. Porewater-corrected sediment compositions, when plotted in Al_2_O_3_, CaO*, Na_2_O and K_2_O space (A-CN-K; where CaO* = Na_2_O when CaO > Na_2_O [molar] and CaO* = CaO when CaO ≤ Na_2_O [molar]; Fedo et al., 1995) also plot close to average upper continental crust (Rudnick and Gao, 2013), but are spread along a weathering trend and display slight K enrichment (Fig. S2B). This can occur with precipitation of K-bearing mineral phases (Fedo et al., 1995).

**S1.2 Redox conditions at the sample sites**

Redox conditions can provide an insight into microbial metabolic processes that operate within sediments. They are also important for understanding the speciation, and thus mobility and bioavailability of chemical elements within fluids. In modern systems it is possible to measure redox proxies like HS^-^ and Eh in fluids directly, but this does not hold for ancient deposits. Thus, we examined redox proxies in addition to hydrothermal signatures preserved within the sediments.

Redox thresholds can be identified in elemental enrichment data from covariance between pairs of variably redox-sensitive elements (Algeo and Li, 2020). All samples are enriched in Mo (Mo_EF_ > 205 ) and U (U_EF_ > 30), with most plotting within a narrow range of values (Fig. S3B). Organic-rich samples plot along a trend that follows roughly three times the typical seawater Mo_EF_/U_EF_ ratio. Samples show enrichments of Re, which is expected as it is more redox sensitive than Mo and U. A slight increase in U_EF_ relative to Re_EF_ occurs in the samples that lacked visible oil migration (*i.e.*, excluding 4870-7) (Fig. S3), which might indicate that the samples with more U were deposited in a weakly reduced setting (where reduced U could accumulate along with reduced Re). However, a lack of co-variance between the Re and U (Fig. S3A) suggest broadly oxic to suboxic conditions. In the oily samples, enrichment of U is evident with respect to Re, without strong covariance. Thus, a different mechanism must be invoked to explain the enrichment of elements like U and Mo (Fig. S3B).

The interpretation of oxic to possibly suboxic conditions is in line with direct measurements from the Southern Trough’s bottom water, where conditions are weakly oxic due to influx of Pacific Deep Water (Campbell and Gieskes, 1984). Such conditions favor the oxidation of ammonium (e.g., by ammonium oxidizing archaea and bacteria) and likely explain why water column ammonium concentrations are generally low away from major hydrothermal plumes in the trough (Campbell and Gieskes, 1984; Lam, 2004). The sedimentary enrichment pattern of molybdenum relative to U_EF_ and seawater (e.g., Algeo & Tribovillard, 2009) matches the expected pattern from an oxic water column, with ‘shuttling’ of Mo that is bound to sinking Fe/Mn particulates. However, the Mo concentrations match more closely the values from black smoker-associated deposits elsewhere in the basin, where Mo is scavenged by hydrothermal sulfide minerals (Eroglu et al., 2020). It is therefore possible that both processes (*i.e.*, Mn-/Fe-oxide shuttling and sulfide scavenging), as well as organic complexation, have played a role in Mo accumulation within the Southern Trough sediments. The enrichment of U and Mo in samples from core 4870-7 (oily samples) is interpreted to reflect complexation and mobilization with organic matter.

**S1.3 Descriptive summary of calculations used (see Supplemental file S2 for details)**

Vertical (one dimensional) steady hydrothermal flow velocities were estimated at half the core depth (20 cm) at three sites with temperature data, using the approach of Bredehoeft & Papaopulos (1965), who provide an analytical solution of the one dimensional advection-diffusion equation by Darcy flow. Darcy flow velocities were then converted to flow velocity using an average sample porosity of 80%, which is representative for all cores and depths. We used thermal conductivity data for Guaymas Basin diatomite sediment from Neumann et al. (2023), which are expected to be applicable here as well. We did not perform the calculation for core 4870-7 due to unknown properties of the hydrocarbon-rich pore fluid (which we were also unable to extract). Flow velocities were then converted to ammonium fluxes using porewater data from samples closest to 20 cm depth in each core. Full details of the calculation and parameters used are presented in Supplemental file S2.

To examine nitrogen loss from sediment in core 4564-13, we fitted an exponential function to the sample TN(wt%) data of the form:

$TN={TN}_{0}*a^{z}$ (1)

where *TN* = total nitrogen content (wt%), *TN_0_* = total nitrogen content at seafloor (wt%), *a* = a loss coefficient and *z* = sample depth. The drop in nitrogen content appears to occur over the top ca. 7cm of sediment, so we imposed a constant (average) TN% for the deeper interval. Equation 1 was integrated and evaluated between the seafloor and the depth at which no further loss was evident (the ‘cutoff z’, ca. 7 cm):

$\int_{seafloor}^{cutoff z} {TN}_{0}\cdot a^{z} dx= {TN}_{0} \frac{a^{cutoff z}-1}{\ln a}$ (2)

Seafloor nitrogen contents were calculated per cm^3^ of porous sediment (using a porosity of 80%), then equation (2) was applied to determine nitrogen loss in the top ca. 7 cm of porous sediment. The lost nitrogen was then converted to a flux using published sedimentation rates of 0.08-0.25 cm/yr (Calvert, 1966; DeMaster, 1981; Geilert et al., 2018). The flux values were then extrapolated across the hydrothermally-active areas of the Southern and Northern Troughs (measured in Google Earth). As an exercise, we examined the impact of the nitrogen flux it were assimilated into biomass in the watercolumn. We assumed steady state, complete vertical mixing and treated the basin as closed. The amount of nitrogen released from the hydrothermal areas was converted to carbon using a C/N molar ratio of 106/16. This value was then divided across the total basin area (measured in Google Earth) to give the amount of C that could be fixed per unit area. For full details of the calculation, please see Supplemental file S2.

**Supplementary figures**


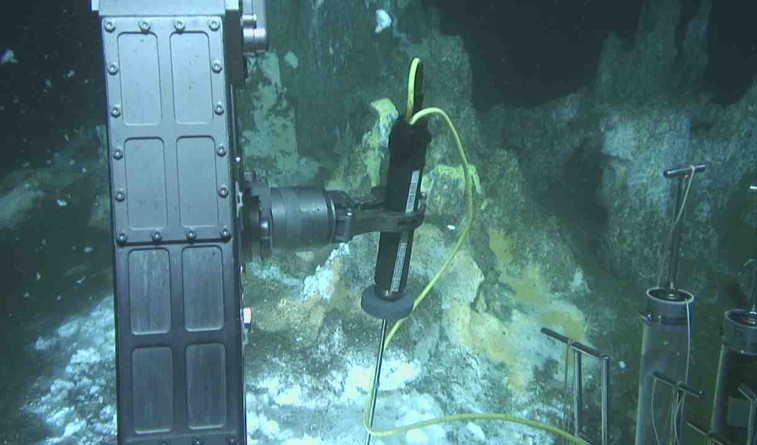


**Figure S1,** temperature probe disturbing white sulfur flocs at the core 4870-7 sample site


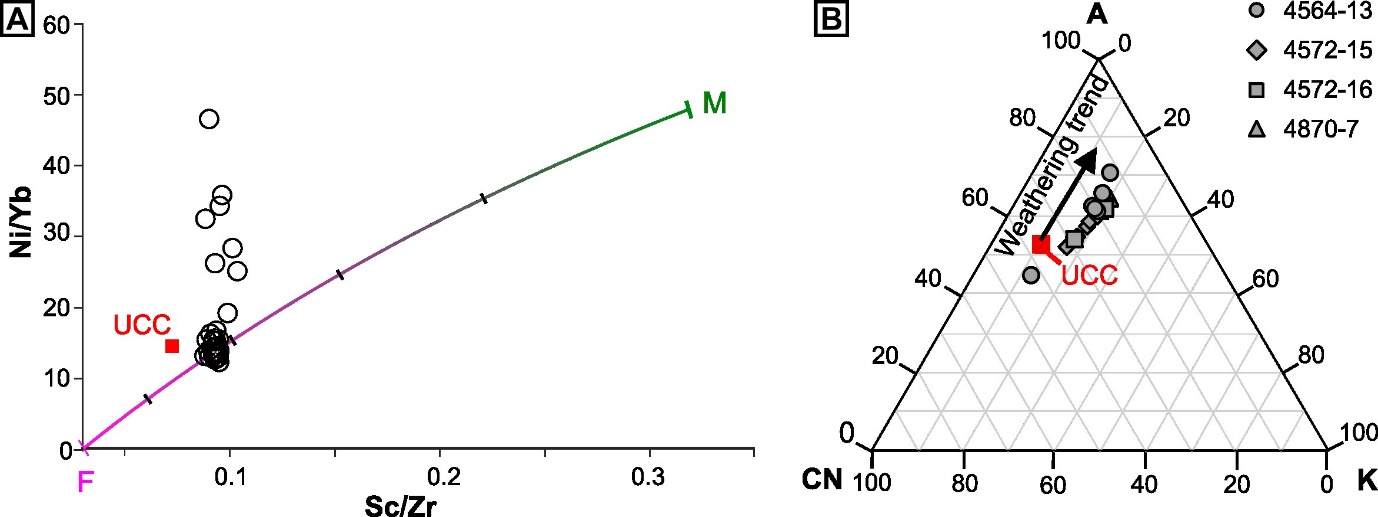
**Figure S2,** sediment provenance and alteration. **A,** Sediment samples and average upper continental crust (UCC; Rudnick & Gao, 2013) plotted along mixing curve between modern felsic (F) and mafic (M) endmember compositions (Ptáček et al., 2020). **B,** A-CN-K ternary plot with a typical weathering trajectory (starting from UCC) indicated (Fedo et al., 1995). Samples are grouped by core and have been corrected for major seawater ions.


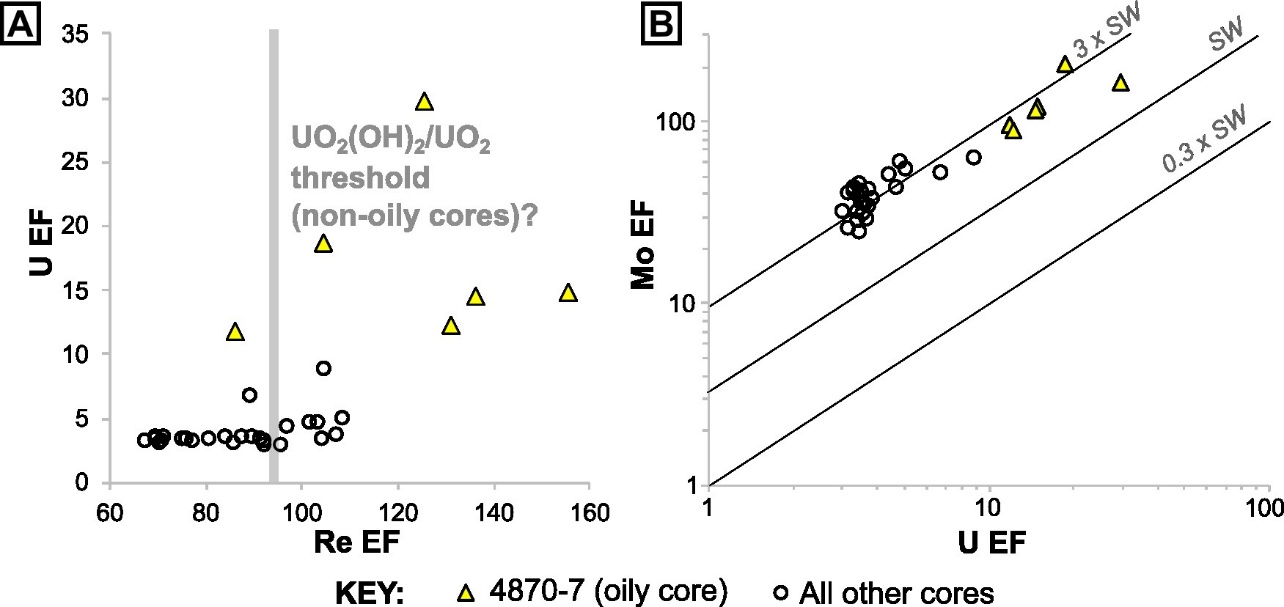
**Figure S3,** redox proxies from elemental enrichment factors (EF) relative to UCC. **A,** a possible subreduced redox threshold (grey line) in non-oily core samples. Weak covariance between U_EF_ and Re_EF_ to the right of the grey line could indicate accumulation of both reduced U and reduced Re in sediments, while the sediments to the left were likely deposited in a more oxic setting (Algeo and Li, 2020). Oily samples are not shown, due to a possible U contribution from migrated organics. **B,** elevated Mo_EF_ and U_EF_ values in the oily and non-oily core samples. Note the greater enrichment in the 4870-7 (oily core) samples. All samples plot at roughly three times the typical seawater Mo_EF_/U_EF_ ratio.


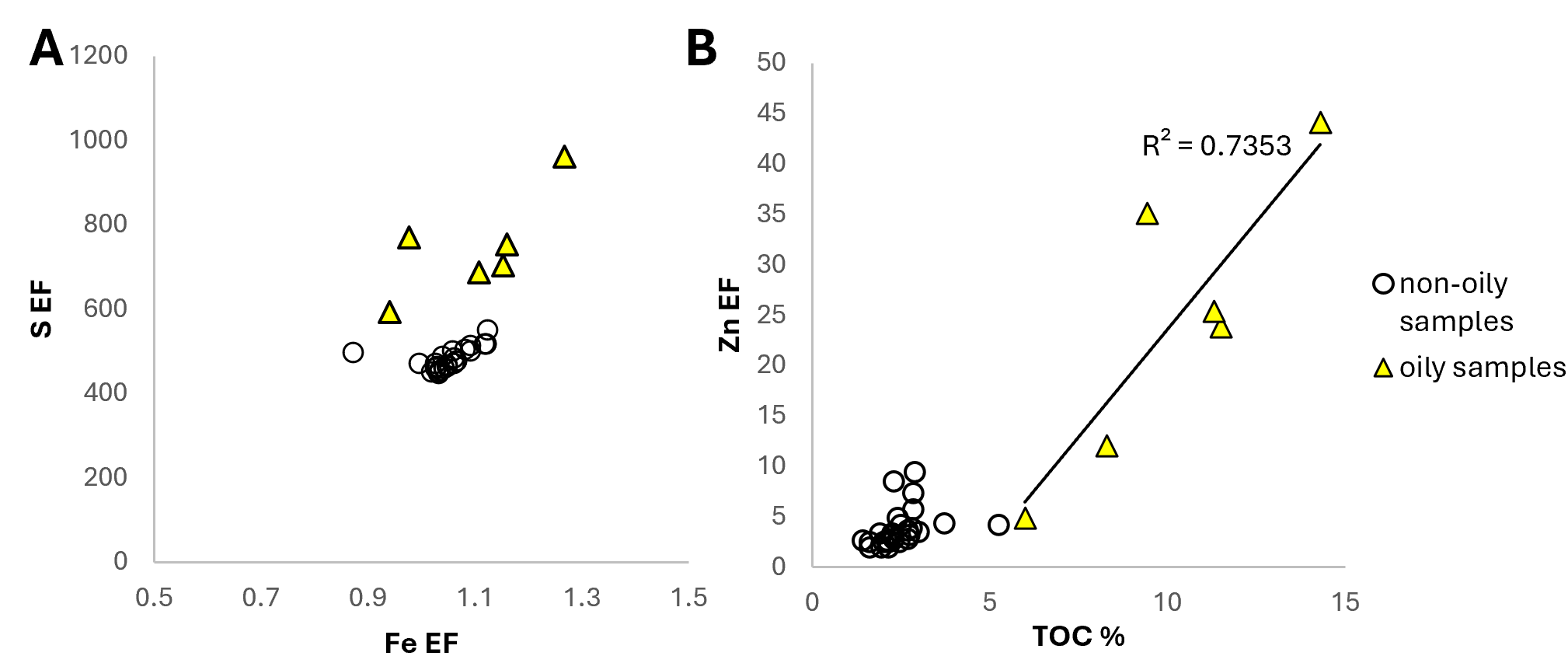


**Figure S4,** additional metal enrichment plots. **A,** covariance between Fe and S enrichment, with oily samples being more enriched overall. **B,** moderate Zn enrichment in oily samples showing covariance with TOC. The highest Zn_EF_ in non-oily cores occurs in deeper samples.


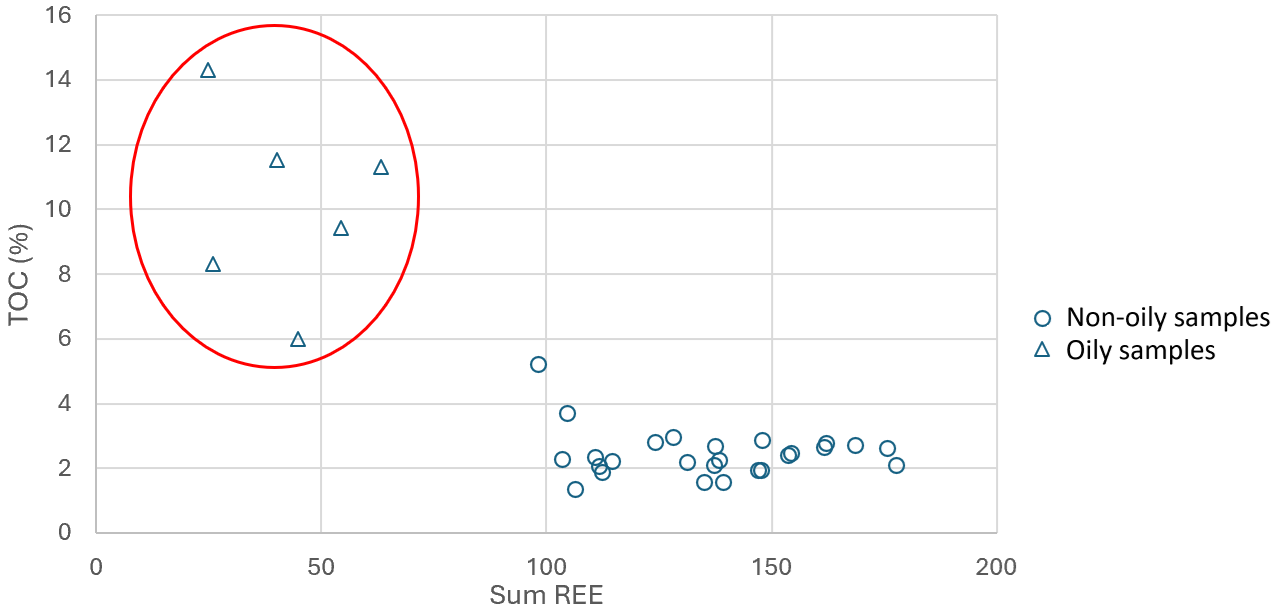


**Figure S5,**  relationship between total organic carbon (TOC) content and total rare earth element content for sediment samples. Oily samples (from core 4870-7) are circled in red.


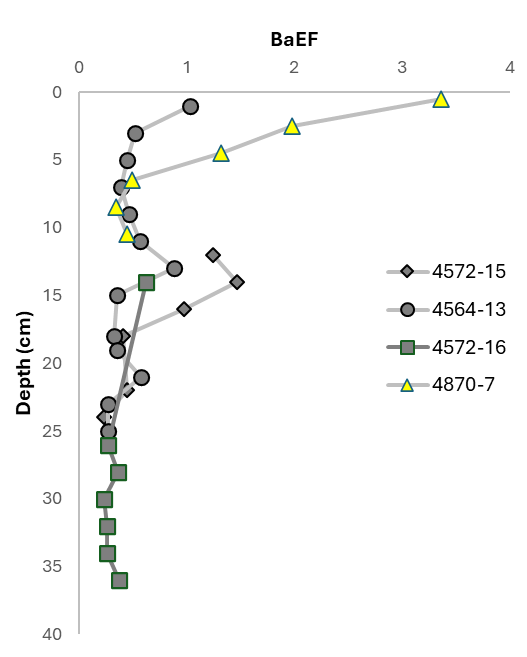


**Figure S6,** Barium enrichment factors in core samples. Note that most of the hotter core samples (4564-13 & 4572-16) are depleted (<1), whereas the hot oily core (4870-7) shows a trend of increasing (albeit slight) enrichment toward the seafloor. Core 4870-7 was taken from an area where barite mineralization has been observed (Witches’ Cauldron). It appears that here, barite is most abundant at the surface – possibly where (seawater?) sulfate and/or barium are most available.

**
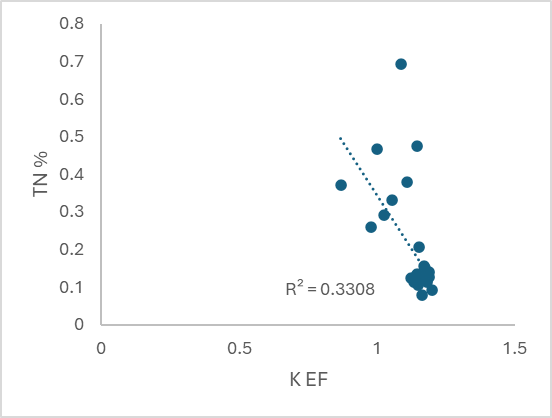
**

**Figure S7,** TN concentration and K enrichment factors for sediment samples (not corrected for contribution from porewater K). Note the results are similar to those in Fig. 5B.


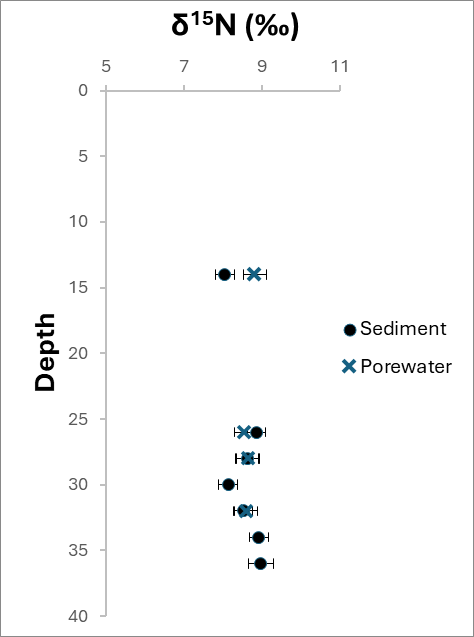


**Figure S8,**  δ^15^N data from sediment and porewater NH_4_^+^ for core 4572-15


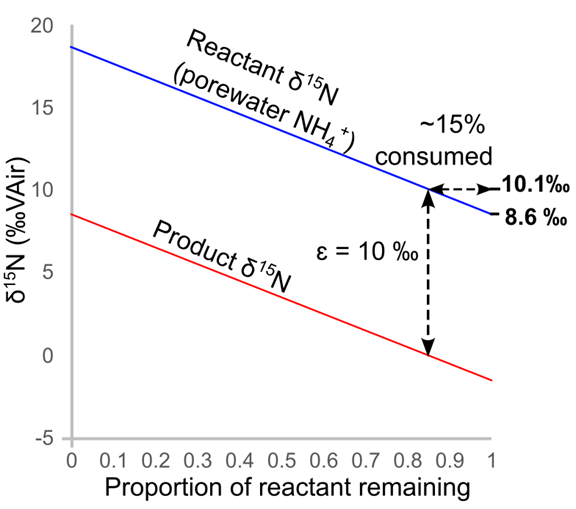


**Figure S9,** steady state model for loss and corresponding isotopic depletion of porewater NH_4_^+^ in core 4564-13, using an initial δ^15^N of 8.6 ‰, final δ^15^N of 10.1 ‰, and consumed fraction of 15%. This approach was used because 4564-13 is from an active seepage site with a consistent supply of reactant NH_4_^+^, which may be partially lost/consumed as it passes upwards through the sediment.

**References**

Algeo, T. J., and Li, C. (2020). Redox classification and calibration of redox thresholds in sedimentary systems. *Geochim Cosmochim Acta* 287. doi: 10.1016/j.gca.2020.01.055

Algeo, T. J., and Tribovillard, N. (2009). Environmental analysis of paleoceanographic systems based on molybdenum-uranium covariation. *Chem Geol* 268. doi: 10.1016/j.chemgeo.2009.09.001

Bredehoeft, J. D., and Papaopulos, I. S. (1965). Rates of vertical groundwater movement estimated from the Earth’s thermal profile. *Water Resour Res* 1. doi: 10.1029/WR001i002p00325

Calvert, S. E. (1966). Accumulation of diatomaceous silica in the sediments of the gulf of California. *Bulletin of the Geological Society of America* 77. doi: 10.1130/0016-7606(1966)77[569:AODSIT]2.0.CO;2

Campbell, A. C., and Gieskes, J. M. (1984). Water column anomalies associated with hydrothermal activity in the Guaymas Basin, Gulf of California. *Earth Planet Sci Lett* 68. doi: 10.1016/0012-821X(84)90140-7

DeMaster, D. J. (1981). The supply and accumulation of silica in the marine environment. *Geochim Cosmochim Acta* 45. doi: 10.1016/0016-7037(81)90006-5

Eroglu, S., Scholz, F., Frank, M., and Siebert, C. (2020). Influence of particulate versus diffusive molybdenum supply mechanisms on the molybdenum isotope composition of continental margin sediments. *Geochim Cosmochim Acta* 273. doi: 10.1016/j.gca.2020.01.009

Fedo, C. M., Wayne Nesbitt, H., and Young, G. M. (1995). Unraveling the effects of potassium metasomatism in sedimentary rocks and paleosols, with implications for paleoweathering conditions and provenance. *Geology* 23. doi: 10.1130/0091-7613(1995)023<0921:uteopm>2.3.co;2

Geilert, S., Hensen, C., Schmidt, M., Liebetrau, V., Scholz, F., Doll, M., et al. (2018). On the formation of hydrothermal vents and cold seeps in the Guaymas Basin, Gulf of California. *Biogeosciences* 15. doi: 10.5194/bg-15-5715-2018

Lam, T. Y. P. (2004). Microbial ammonia oxidation in deep-sea hydrothermal plumes. University of Hawai’i at Manoa.

Neumann, F., Negrete-Aranda, R., Harris, R. N., Contreras, J., Galerne, C. Y., Peña-Salinas, M. S., et al. (2023). Heat flow and thermal regime in the Guaymas Basin, Gulf of California: Estimates of conductive and advective heat transport. *Basin Research*. doi: 10.1111/bre.12755

Ptáček, M. P., Dauphas, N., and Greber, N. D. (2020). Chemical evolution of the continental crust from a data-driven inversion of terrigenous sediment compositions. *Earth Planet Sci Lett* 539. doi: 10.1016/j.epsl.2020.116090

Rudnick, R. L., and Gao, S. (2013). “Composition of the Continental Crust,” in *Treatise on Geochemistry: Second Edition*. doi: 10.1016/B978-0-08-095975-7.00301-6
